# Association between mucus phenotypes, histopathological features, and ultra-high magnification endoscopic findings in ulcerative colitis

**DOI:** 10.1038/s41598-026-53592-3

**Published:** 2026-05-22

**Authors:** Takeshi Hashimoto, Hiroki Kurumi, Shungo Kanetsuki, Tsutomu Kanda, Yuichiro Ikebuchi, Koichiro Kawaguchi, Kazuo Yashima, Tomohiko Sakabe, Yoshihisa Umekita, Hajime Isomoto

**Affiliations:** 1 Division of Gastroenterology and Nephrology, Department of Multidisciplinary Internal Medicine, Faculty of Medicine, Tottori University, Tottori, Japan; 2 Department of Pathology, Faculty of Medicine, Tottori University, Tottori, Japan

**Keywords:** Ulcerative colitis, Mucus phenotype, Mucin, MUC5AC, Endocytoscopy, Diseases, Gastroenterology

## Abstract

Ulcerative colitis (UC) is a chronic inflammatory bowel disease affecting the colonic mucosa. Changes in mucin phenotype may be associated with mucosal inflammation in UC. However, the relationship between clinicopathological features and mucus phenotypes in UC remains unclear. Therefore, we aimed to evaluate the association between mucus phenotypes and clinicopathological features in patients with UC using immunohistochemistry, as well as their association with ultra-high magnification endocytoscopy (EC) classification. A total of 51 lower gastrointestinal endocytoscopy procedures were performed for 39 patients with UC between 2018 and 2022. MUC2 expression was positive in all biopsy specimens, whereas MUC1, MUC6, and CD10 expression was negative. MUC5AC was expressed in 51.3% of cases. MUC5AC positivity was associated with duration of disease, classification by clinical course, Mayo score, Mayo endoscopic subscore (MES), Nancy histological index, C-reactive protein levels, erythrocyte sedimentation rate, and EC classification at the patient level. At the specimen level, significant differences were observed in the Nancy histological index, MES, and EC classification. In multivariate analysis, the Nancy histological index showed a significant correlation.MUC5AC is strongly correlated with histopathological severity and may also be associated with clinical and endoscopic severity. The clinical significance of MUC5AC positivity remains a topic for future investigation.

**Supplementary Information:**

The online version contains supplementary material available at 10.1038/s41598-026-53592-3.

## Introduction

Ulcerative colitis (UC) is an intractable intestinal disease that causes diffuse inflammation mainly in the colonic mucosa. Ulcerative colitis, along with Chron’s disease, is classified as inflammatory bowel disease (IBD)^1^. The exact etiology of UC is unknown; however, it is a multifactorial disease involving genetics, environmental factors, luminal factors, and intestinal microbiota, all of which interact to induce an excessive immune response, developing IBD^2,3^.

Changes in the mucus lining of the intestinal tract in patients with UC may be associated with intestinal inflammation^4^. Mucin (MUC), the main component of mucus secreted by intestinal mucosal epithelial cells, is a high-molecular-weight glycoprotein with properties that vary depending on the site within the digestive tract. There are two categories of MUC, membrane-bound and secreted, and at least 20 types have been reported. In a normal colon, MUC2 is the most secreted glycoprotein and is produced by goblet cells^5^. MUC glycoproteins are secreted into the gastrointestinal lumen and play a role in protecting the intestinal mucosa, preventing direct bacterial invasion and selectively absorbing substances. Mice lacking MUC2 spontaneously have been reported to develop colitis, suggesting an association between mucus characteristics and colitis^6,7^.

In addition, there have been reports on cases in which gastric-type mucus trait markers such as MUC5AC (gastric orbital epithelial type) and MUC6 (gastric pyloric gland type) were expressed in the inflamed mucosa of patients with UC. These were particularly frequent in UC-associated colorectal cancer and its precancerous lesion, dysplasia^8,9^. It has also been suggested that a decrease in mucin is associated with future relapse, and the importance of mucus function is an area of increasing interest^10,11^.

Therefore, changes in mucous phenotypes may be associated with intestinal inflammatory activity and inflammatory carcinogenesis; however, the relationship between clinicopathological features and mucous phenotypes in patients with UC has not been fully elucidated. In this study, we aimed to evaluate mucus traits in the colonic mucosa of patients with UC using immunohistochemistry and examine their association with clinicopathological features. We previously proposed the “Endocytoscopy (EC) classification,” as EC can magnify the mucosa up to 400–1000x. EC evaluates the degree of inflammation by real-time observation of the colonic mucosa of patients with UC at the cellular and nuclear levels, and we found that the classification correlates with histopathological findings^12^. Therefore, we also aimed to examine the relationship between EC classification and mucus traits.

## Materials and methods

### Study population

This study was a single-center observational study. Thirty-nine patients aged 20 years or older with UC who were attending the Department of Gastroenterology, Tottori University Hospital were enrolled in this study. The diagnosis of UC was based on clinical, laboratory, endoscopic, histopathologic, and radiologic parameters^13^. The study was conducted in accordance with the Declaration of Helsinki and approved by the Ethics Committee of Tottori University Hospital (approval number: 1512A094). Written informed consent was obtained from all patients.

### Methods

**Fig. 1:**
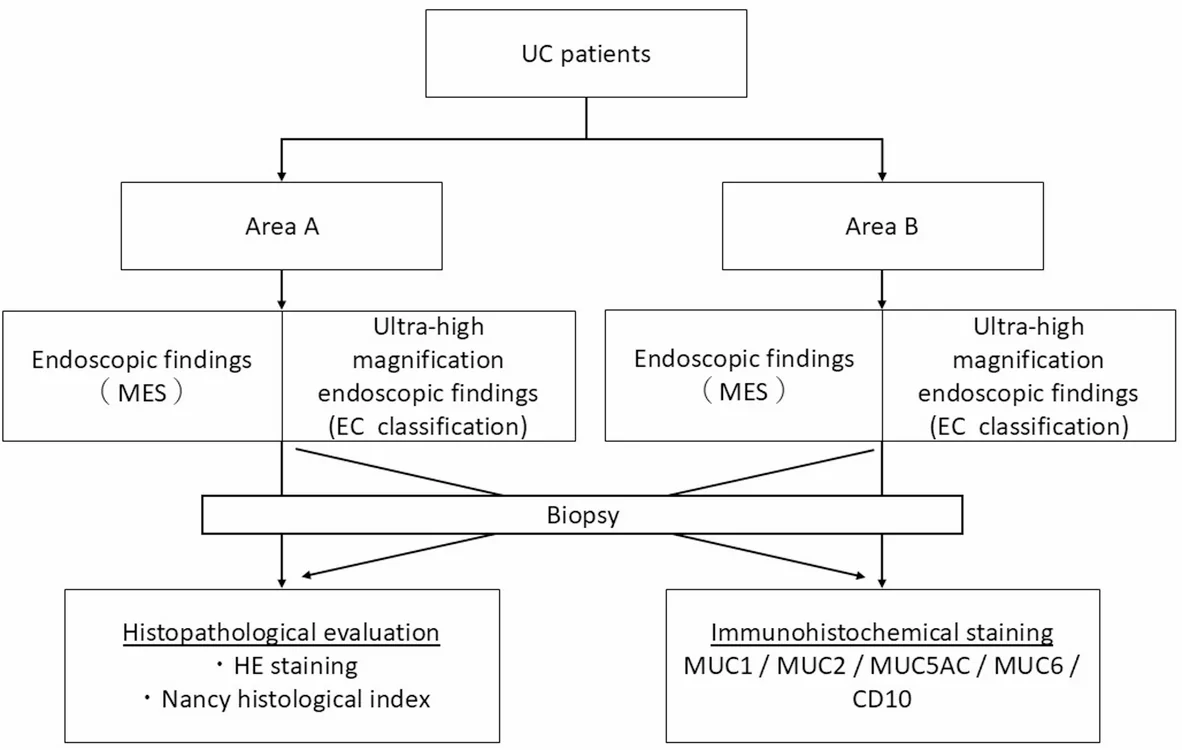
Flowchart of endoscopic observation, biopsy, fixation, and staining. UC, ulcerative colitis; MES, Mayo endoscopic subscore; EC, endocytoscopic classification; HE, hematoxylin-eosin; MUC, mucin; CD, cluster of differentiation.

**Fig. 2:**
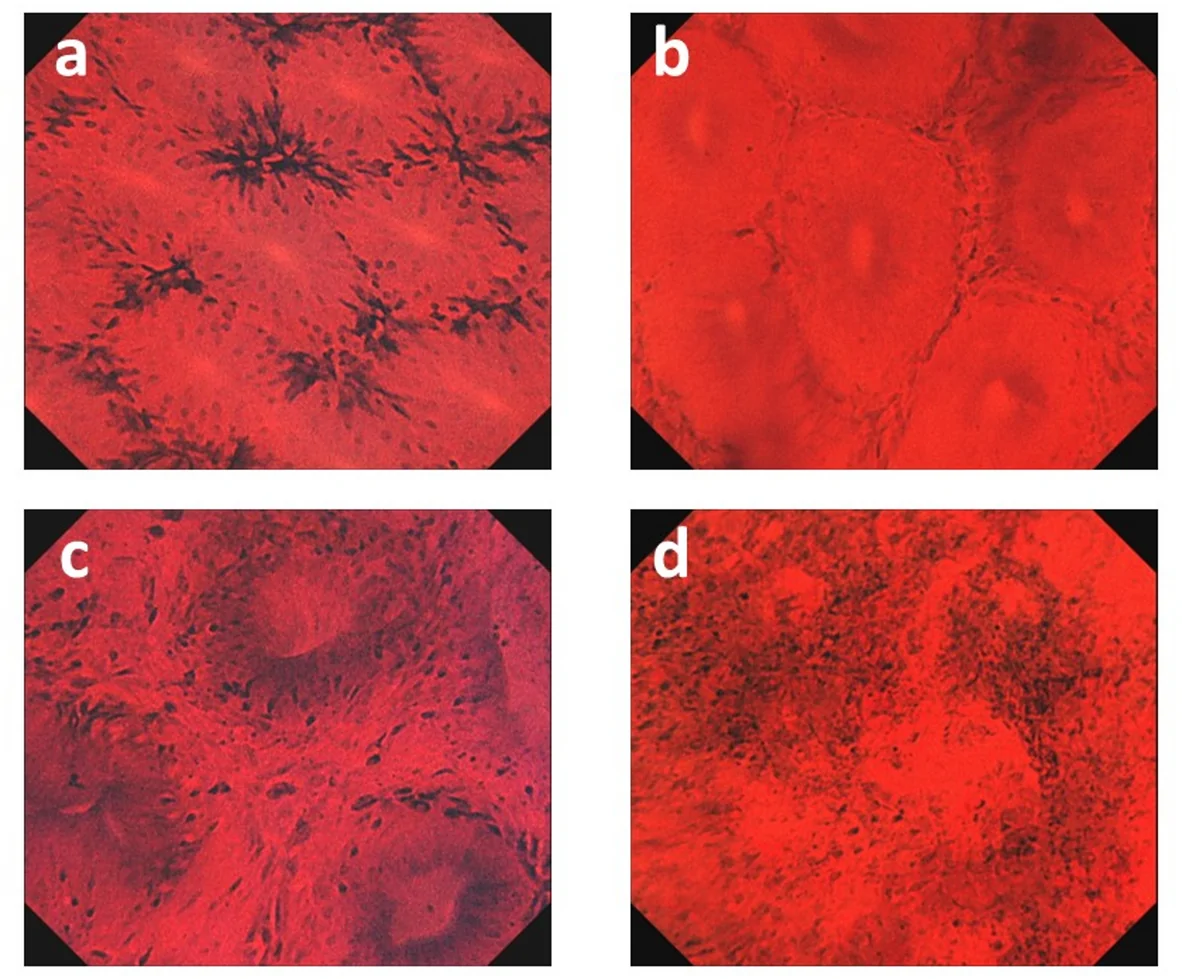
Reference image of EC classification. (**a**) EC-A; regular arrangement of round to oval pits. (**b**) EC-B; irregular arrangement with/without enlarged spaces between regular pits. (**c**) EC-C; deformed pits with distorted crypt lumen which are unordered in arrangement but not disrupted. (**d**) EC-D; disruptive or disappeared pits.

We analyzed 51 lower gastrointestinal endoscopic procedures performed between April 2018 and May 2022 using an EC (OLYMPUS GIF TYPE H-290EC or OLYMPUS CF TYPE H-290ECI, Olympus Co., Tokyo, Japan), which allows magnification up to 520x. This study is a retrospective study using existing data and samples. Samples were collected from two sites: the area with the highest endoscopic disease activity (Area A) and the area with the lowest endoscopic activity or no activity (Area B). The endoscopic severity of each area was assessed using the Mayo Endoscopic Subscore (MES)^14^. Mucosal staining was performed by either spraying both 0.05% crystal violet and 1% methylene blue, or 1% methylene blue alone, and the mucosal microstructure was assessed by ultra-high magnification observation. The microstructure of the mucosa was evaluated using the EC classification (Ueda classification)^12^, and biopsies were obtained from the EC-classified sites (Fig. 1). The EC classification (Ueda classification) classifies them into four types, EC-A to D, and defines them as follows: EC-A, regular arrangement of round to oval pits; EC-B, irregular arrangement with/without enlarged spaces between regular pits; EC-C, deformed pits with distorted crypt lumen which are unordered in arrangement but not disrupted; and EC-D, disruptive or disappeared pits (Fig. 2).

**Fig. 3:**
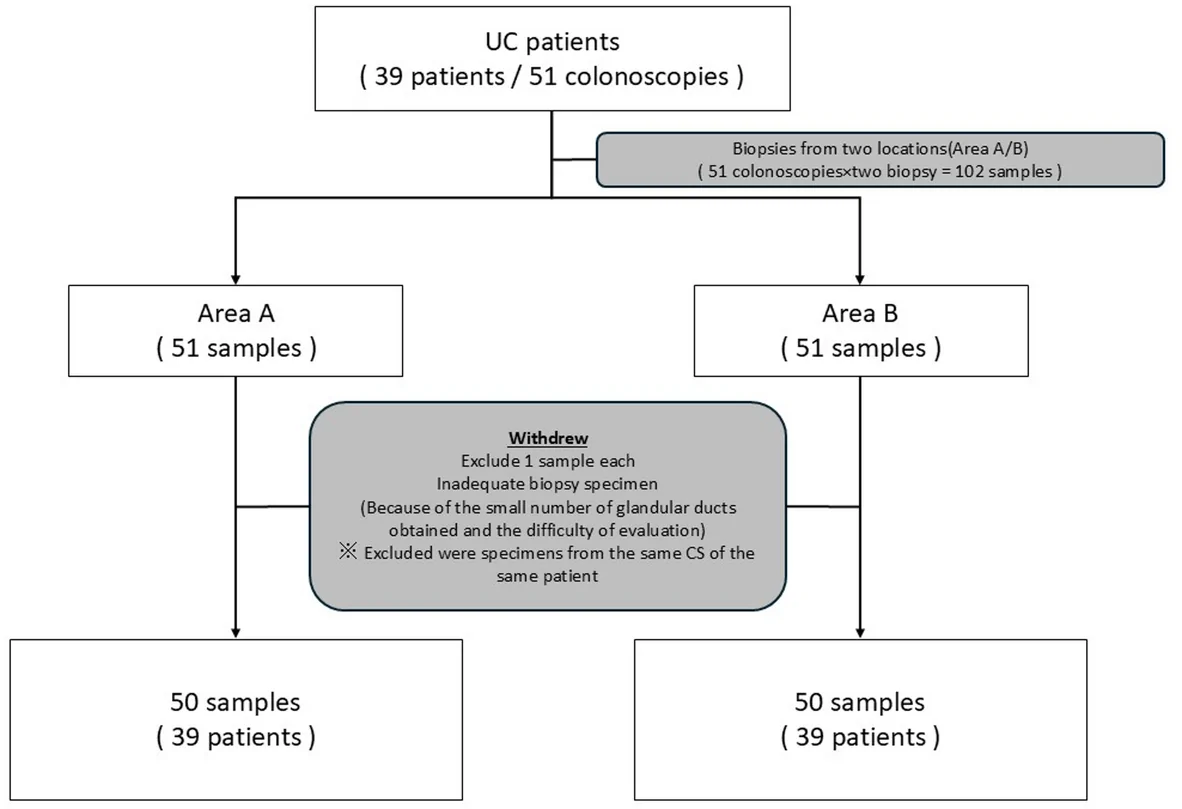
Number of patients and sample size. One case and two samples were excluded from the study owing to inappropriate specimens. CS, colonoscopy; UC, ulcerative colitis.

A total of 102 colorectal mucosal tissue samples were collected via biopsy from both Area A and B during 51 lower gastrointestinal endoscopies performed on 39 patients. These samples were evaluated histopathologically and immunohistochemically. Of the 39 patients, 10 underwent two endoscopic examinations and one underwent three endoscopic examinations at different times, with samples collected at each examination. For one of the patients who underwent two examinations, the number of glandular ducts required for a proper immunohistochemical assessment was insufficient in one of the examinations; therefore, the two samples collected during that examination were excluded, resulting in 100 samples being included in the final analysis (Fig. 3).

Clinicopathological data including sex, age, disease duration, Montreal classification for UC^15^, classification by clinical course^16^, Mayo score^14^, current treatment, blood test findings (C-reactive protein level, erythrocyte sedimentation rate, and white blood cell count), MES, EC classification (Ueda classification), tissue collection site, and Nancy histological index^17^ were collected and analyzed in relation to mucous morphology. Classification by clinical course is divided into four types: first attack type, relapse-remitting type, chronic continuous type, and acute fulminating type. The first attack type refers to cases showing activity only at onset and achieving remission within 6 months. The relapse-remitting type involves alternating active and remission phases. The chronic continuous type involves being in the active phase for 6 months or longer from onset. The acute fulminating type is defined by extremely severe symptoms at onset and a critical condition. A Mayo score of 0–2 was defined as remission, 3–5 as mild active disease, 6–10 as moderate active disease, and 11–12 as severe active disease. Four endoscopy specialists evaluated the endoscopic findings, including MES and EC classification, for all cases.

Furthermore, the 100 collected samples were divided into inflammatory and non-inflammatory areas, defining inflamed areas as those with a MES score of 1 or higher, and subjected to additional analysis. Additionally, areas that had ever exhibited inflammation due to ulcerative colitis were defined as lesional areas, while those that had never exhibited such inflammation were defined as non-lesional areas, and the relationship with inflammation was also examined.

### Immunohistochemistry

Tissues obtained from biopsies were fixed in formalin and embedded in paraffin to create blocks, which were then cut into 3–4 μm-thick paraffin sections. These sections were then immersed in antigen activation solution at a pH of 9 and heated at 100 °C for 20 min in HEAT PRO II (NICHIREI BIOSCIENCES INC.).

For immunohistochemical examination, immunostaining was performed using an automated immunostainer (HISTOSTAINER 36 A; NICHIREI BIOSCIENCES Inc.) according to the manufacturer’s protocol. The five primary antibodies MUC1 (Ma695; Ready-to-Use, Leica Biosystems Newcastle Ltd, Newcastle upon Tyne NE12 8EW, United Kingdom), MUC2 (Ccp58; Ready-to-Use, Leica Biosystems Newcastle Ltd, Newcastle upon Tyne NE12 8EW, United Kingdom), MUC5AC (CLH2; Ready-to-Use, Leica Biosystems Newcastle Ltd, Newcastle upon Tyne NE12 8EW, United Kingdom), MUC6 (CLH5; Ready-to-Use, Leica Biosystems Newcastle Ltd, Newcastle upon Tyne NE12 8EW, United Kingdom), and CD10 (56C6; Ready-to-Use, NICHIREI BIOSCIENCES INC., Tokyo, Japan) were used. In addition, staining was also performed with the corresponding isotype control antibody (ab18443; Mouse IgG1, kappa monoclinal [MOPC-21], Abcam, Cambridge, United Kingdom) to confirm isotype control staining.

**Fig. 4:**
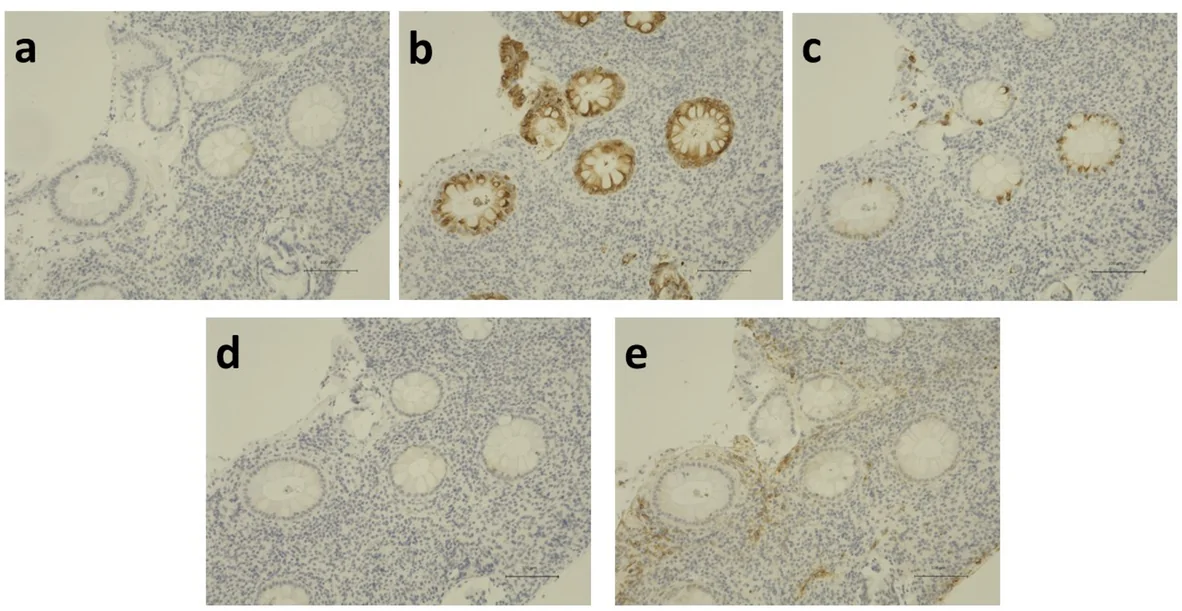
Biopsy specimen from Area A. Immunostaining of the same samples. (**a**) MUC1, (**b**) MUC2, (**c**) MUC5AC, (**d**) MUC6, and (**e**) CD10; stained with CD10 lymphocytes. Images were obtained using the 20× objective lens of a microscope (BZ X710, KEYENCE Corp., Osaka, Japan) connected to a digital camera and saved as images (size 1920 × 1440 pixels). MUC, mucin; CD, cluster of differentiation.

Immunostained pathological specimens were evaluated by two gastroenterologists and one pathologist, and they were considered positive if more than 1% of the glandular duct cells in the biopsy tissue were positive^18^ (Fig. 4).

### Statistical analysis

Data collected were first assessed for normality using the Shapiro–Wilk test. Because the data did not follow a normal distribution, nonparametric methods were employed for the analyses. All continuous variables were non-normally distributed and are presented as medians with interquartile ranges. To compare categorical variables such as EC classification (EC-A/B/C/D) and immunostaining results (MUC1, MUC2, MUC5AC, MUC6, CD10), the Fisher’s exact test was applied. MUC5AC-positive and MUC5AC-negative groups were also compared using the Fisher’s exact test for categorical variables including sex, Montreal classification for UC, biopsy site and clinical course. For continuous variables, including age, disease duration, Mayo score, CRP level, erythrocyte sedimentation rate (ESR), white blood cell (WBC) count, Nancy histological index, and MES, the Mann–Whitney U test was used, as the data were unpaired and non-normally distributed. After conducting univariate analysis, a multivariate logistic regression analysis was performed. All statistical analyses were conducted using SPSS Statistics version 27 (IBM Corp., Armonk, NY, USA), and a p-value < 0.05 was considered statistically significant.

## Results

### Patient characteristics

This study included 24 males and 15 females, and for patients who underwent multiple tests, calculations were performed using only the first data, with the results shown in the table (Table 1). The median age at the time of endoscopy was 45 years (interquartile range(IQR) : 37.0–60.0), and the median disease duration of 63.0 months (IQR: 25.5-115.5). The extent of inflammation was classified as E1 (proctitis) (*n* = 9/39), E2 (left-sided colitis) (9/39), and E3 (extensive colitis) (21/39) according to the Montreal classification for UC. Regarding clinical course at the time of endoscopy, 20 cases were classified as first-attack type, 16 as relapsing–remitting type, and 3 as chronic persistent type; no cases were classified as acute fulminant type.

**Table 1:** Patient characteristics of the 39 patients analyzed.

PatientCharacteristics	n = 39
Sex (n, %)	
Male	24 (61.5)
Female	15 (38.5)
Age, median (IQR)	45.0 (37.0–60.0)
Duration of disease (month), median (IQR)	63.0 (25.5–115.5)
Montreal classification for UC (n, %)	
E1	9 (23.1)
E2	9 (23.1)
E3	21 (53.8)
Classification by clinical course (n, %)	
First attack type	20 (51.3)
Acute fulminating type	0 (0)
Relapse-remitting type	16 (41.0)
Chronic continuous type	3 (7.7)
Mayo score, median (IQR)	1 (0–3)
Remission (n, %)	27 (69.2)
Mild (n, %)	9 (23.1)
Moderate (n, %)	3 (7.7)
Severe (n, %)	0 (0)
Treatment	
5-ASA (n, %)	33 (84.6)
PSL (n, %)	5 (12.8)
GMA (n, %)	0 (0)
IM (n, %)	12(30.8)
Bio (n, %)	1 (2.6)
CRP, mg/L, median (IQR)	0.05 (0.02–0.10)
ESR, mm, median (IQR)	12 (5–18)
WBC, /μL, median (IQR)	5400 (4950–6250)
MES, median (IQR)	1 (0–2)
0 (n, %)	13 (33.4)
1 (n, %)	12 (30.8)
2 (n, %)	13 (33.4)
3 (n, %)	1 (2.6)

UC, ulcerative colitis; 5-ASA, 5-aminosalicylic acid; PSL, prednisolone; Bio, Biologics; GMA, Granulocyte and Monocyte Apheresis; IM, immunomodulator; IQR, interquartile range.

Montreal classification for UC: E1 = proctitis, E2 = left-side colitis, E3 = extensive colitis.

Mayo score: remission = 0–2, mild = 3–5, moderate = 6–10, and severe = 11–12.

The median Mayo score was 1. Based on the Mayo score, 27 cases were classified as being in remission, 9 in the mildly active stage, 3 in the moderately active stage, and none in the severely active stage. Endoscopic severity, as assessed by MES, was 0 in 13 cases, 1 in 12 cases, 2 in 13 cases, and 3 in 1 cases. The therapeutic agents used at the time of endoscopy were 5-ASA for 33 patients, corticosteroids for 5 patients, immunomodulators for 12 patients, and biologic agents for 1 patients. The median blood test findings were CRP 0.05 mg/L (IQR: 0.02–0.10), ESR 12 mm (IQR: 5–18), and WBC 5400 /µL (IQR: 4950–6250) (Table 1).

Biopsy specimens were collected from sites first evaluated using MES, followed by EC classification and tissue sampling. The biopsy sites included the ileum (*n* = 6), ascending colon (*n* = 14), transverse colon (*n* = 10), descending colon (*n* = 13), sigmoid colon (*n* = 14), and rectum (*n* = 43). The local MES at each biopsy site was 0 in 59 cases, 1 in 23 cases, 2 in 15 cases, and 3 in 3 cases. The EC classification at these sites was EC-A in 56 cases, EC-B in 29, and EC-C/D in 15. The Nancy histological index of the collected specimens was grade 0 in 55 cases, grade 1 in 16 cases, grade 2 in 20 cases, grade 3 in 9 cases, and no grade 4.

### Immunohistochemistry

**Fig. 5:**
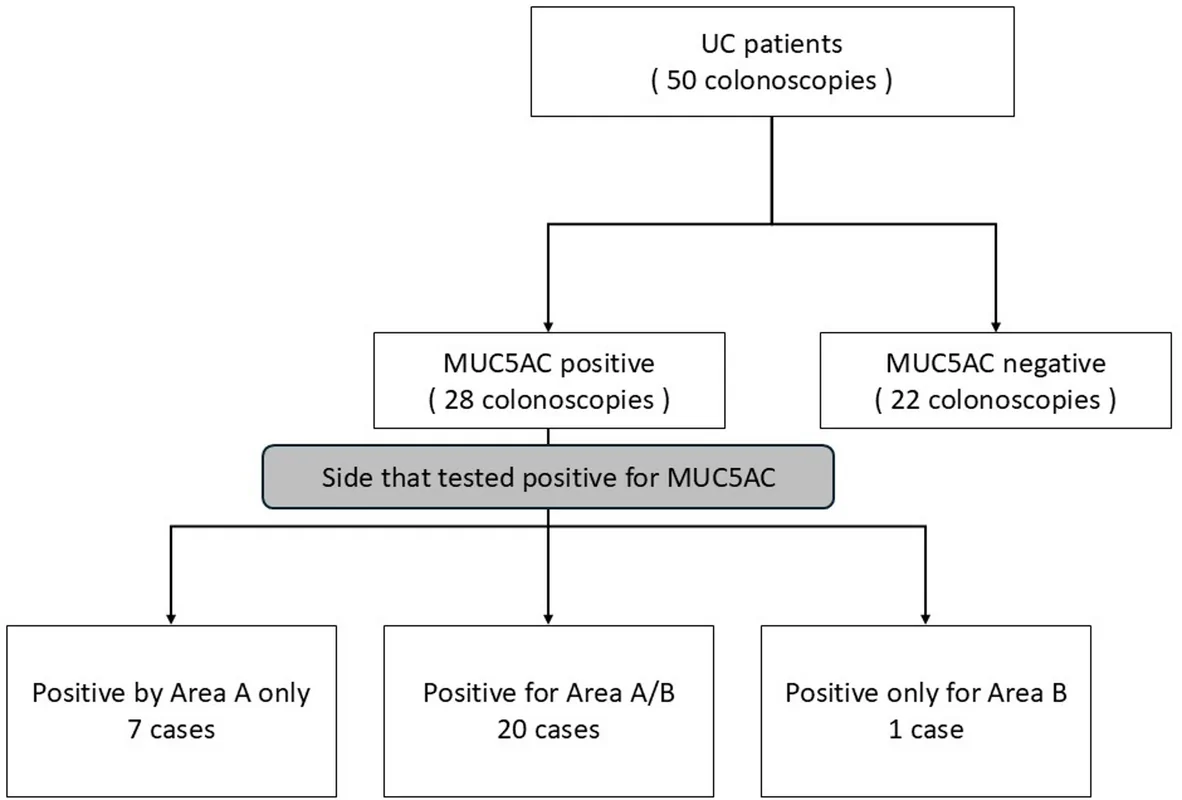
MUC5AC positive and negative groups. Breakdown of the MUC5AC positive group. UC, ulcerative colitis; MUC, mucin.

Immunostaining was performed for MUC1, MUC2, MUC5AC, MUC6, and CD10. All specimens were negative for MUC1, MUC6, and CD10, but positive for MUC2. It was confirmed that there was no staining in the isotype control staining. Additionally, MUC5AC was positive in 51.3% (20/39) of the patients. Endoscopy revealed that 56.0% (28/50) of the procedures yielded MUC5AC-positive samples: 20 in the Area A only, 7 in both Area A and B, and 1 in the Area B only (Fig. 5). When analyzed by sample, MUC5AC was positive in 35% (35/100) of the samples.

Based on these results, subsequent analyses were focused on MUC5AC.

### Comparison of clinical features and mucus phenotype

The endoscopy group from which MUC5AC-positive specimens were obtained was defined as the MUC5AC-positive endoscopy group (5AC positive-endo). In univariate analysis, the 5AC positive-endo group showed a significantly higher proportion of the relapse-remitting type. Furthermore, duration of disease, Mayo score, CRP, ESR, MES, and Nancy histological index were significantly higher in the 5AC positive-endo group than in the MUC5AC-negative endoscopy group (5AC negative-endo). However, no significant differences were observed between the groups regarding age, sex, Montreal classification for UC, or WBC count.

In the multivariate analysis, significant differences were observed in the classification by clinical course. Laboratory data (CRP, ESR, WBC) were not included in the multivariable analysis because most patients had mild disease, resulting in limited variation in these parameters. In addition, the small sample size made stable estimation difficult, and some laboratory variables showed unstable estimates (Table 2).

**Table 2:** Properties of MUC5AC-positive and MUC5AC-negative endoscopy groups.

Patient characteristics	MUC5AC positive-endo	MUC5AC negative-endo	Univariate analysis		Multivariate analysis	
			*p value*	OR	95%CI	*p value*
Sex						
Male	17	14	*p* = 1	1.497	0.322–6.955	0.607
Female	11	8				
Age, median (IQR)	46.5(38.0–63.3)	44.5(32.3–52.5)	*p* = 0. 5	0.948	0.928–1.053	0.714
Duration of disease (month), median (IQR)	105 (62–188.5)	43(18–108)	*p* = 0.0127	1.008	0.997–1.020	0.155
Montreal classification for UC						
E1	4	5	*p* = 0.509			
E2	10	5				
E3	14	12				
Classification by clinical course						
First attack type	8	15	*p* = 0.00321	1.78	1.037–3.055	0.036
Acute fulminating type	0	0				
Relapse-remitting type	18	3				
Chronic continuous type	2	4				
Mayo score, median (IQR)	2.0 (1.0–4.0)	0(0–1.0)	*p* = 0.00559	1.074	0.684–1.685	0.756
Treatment						
5-ASA	25	19				
PSL	2	4				
GMA	0	0				
IM	13	2				
Bio	5	0				
CRP, mg/L, median (IQR)	0.07(0.02–0.1625)	0.03(0.022–0.05)	*p* = 0.0447			
ESR, mm, median (IQR)	12(8–19)	5(3.0–12.0)	*p* = 0.0156			
WBC, /μL, median (IQR)	5650(4875–6200)	5300(4825–6050)	*p* = 0.261			
MES, median (IQR)	1.5 (1–2)	0.5 (0–1)	*p* = 0.00562	1.437	0.393–5.258	0.584
0	6	11				
1	8	8				
2	12	3				
3	2	0				
Nancy histological index, Grade, median (IQR)	2 (1.00–2.25)	0 (0.00–1.75)	*p* = 0.0112	1.559	0.690–3.521	0.286

Cases that were MUC5AC positive only in Area Bwere statistically analyzed using data from Area B.

IQR, interquartile range; UC, ulcerative colitis; 5-ASA, 5-aminosalicylic acid; PSL, prednisolone; GMA, Granulocyte and Monocyte Apheresis; IM, immunomodulator; Bio, Biologics; CRP, C-reactive protein; ESR, erythrocyte sedimentation rate; WBC, white blood cell; MES, Mayo endoscopic subscore.

Montreal classification for UC: E1 = proctitis, E2 = left-side colitis, E3 = extensive colitis.

### Comparison of endoscopic features and mucus phenotype

When analyzed by sample, MUC5AC-positive biopsy specimens were classified as the MUC5AC-positive sample group (5AC positive-sam). Univariate analysis showed that the 5AC-positive sample group had higher local MES, Nancy histological index, and EC classification compared to the 5AC-negative sample group at the biopsy site. However, no significant difference was observed in the MUC5AC positivity rate at colon biopsy sites. Multivariate analysis revealed that the Nancy histological index was significantly higher in the 5AC-positive sample group compared to the 5AC-negative sample group. While a significant difference was observed in EC classification in univariate analysis, no significant difference was found between the two groups in multivariate analysis.

Based on current mucosal inflammation, samples were classified as inflammatory area (MES ≥ 1) and non-inflammatory area (MES 0). 41 samples were obtained from inflammatory areas, and 59 from non-inflammatory areas. The MUC5AC positivity rate was significantly higher in inflammatory areas.

Furthermore, we defined areas that had previously been inflamed as lesional area and areas that had not been inflamed as non-lesional area for our analysis. 82 samples were obtained from lesional areas, and 18 samples were obtained from non-lesion sites. No significant difference in MUC5AC was observed between lesion sites and non-lesion sites (Tables 3 and 4).

**Table 3:** Correlation of MUC5AC staining with biopsy site, biopsy Nancy histological index, MES, and EC classification.

	MUC5AC positive sam (n = 35)	MUC5AC negative sam (n = 65)	Univariate analysis		Multivariate analysis	
			*p value*	OR	95%CI	*p value*
Biopsy site						
Ileocecum	0	6	*p* = 0.104	1.007	0.746–1.360	0.963
Ascending colon	8	6				
Transverse colon	6	4				
Descending colon	5	8				
Sigmoid colon	5	9				
Rectum	11	32				
Nancy histological index, Grade, median (IQR)	2 (0.5–2)	0 (0–1)	*p* = 0. 00000196	1.891	1.051–3.403	0.034
MES						
0	10	49	*p* = 0. 00000131	1.715	0.684–4.300	0.25
1	11	12				
2	12	3				
3	2	1				
EC classification						
A	11	45	*p* = 0. 0000561	1.537	0.586–4.034	0.449
B	12	17				
C/D	12	3				
Inflammation of biopsy site						
Inflammatory area (MES ≧ 1)	25	16	*p* = 0. 0000123			
Non-inflammatory area (MES 0)	10	49				
Lesion or non-lesional area of biopsy site						
Lesional area	31	51	*p* = 0. 279			
Non-lesional area	4	14				

IQR, interquartile range; MES, mayo endoscopic subscore.

**Table 4:** Characteristics of mucus by EC classification.

		MUC1	MUC2	MUC5AC	MUC6	CD10
EC classification	EC-A (n = 56)	0/56 (0%)	56/56 (100%)	11/56 (19.6%)	0/56 (0%)	0/56 (0%)
	EC-B (n = 29)	0/29 (0%)	29/29 (100%)	12/29 (41.4%)	0/29 (0%)	0/29 (0%)
	EC-C/D (n = 15)	0/15 (0%)	15/15 (100%)	12/15 (80.0%)	0/15 (0%)	0/15 (0%)
*p value*				*p* = 0.0000561		

Specimens from both lesional and non-lesional areas were evaluated using the EC classification, and immunostaining was performed for each EC classification class.

## Discussion

The human gastrointestinal tract produces a characteristic mucus, which serves various functions. In the large intestine, the mucus layer acts as the first barrier on the surface of the digestive tract, effectively isolating intestinal epithelial cells from the intestinal lumen and protecting them from contact and irritation by intestinal bacteria, their metabolites, and food antigens^19^. The mucus layer of the colon comprises approximately 30 core proteins, including MUCs, antimicrobial peptides, and secreted immunoglobulin A. Among them, MUC2, which is synthesized in goblet cells, is the most prominent MUC expressed in the colons of healthy individuals and in patients with UC^5^. However, biopsy samples have revealed that the mucus layer in patients with UC is thinner, particularly in areas of inflammation^20^. Therefore, the reduction in goblet cell numbers associated with mucosal damage in UC may be related to decreased MUC expression^5^. This is associated with loss of mucosal barrier integrity, which can lead to microbial infiltration of the mucosa, increasing contact between the epithelium and microflora, and may further promote inflammation^21^. In contrast, Van Klinken et al. reported increased MUC2 sulfation in patients with UC^22^. Sulfation of MUC sugar chains is considered an essential modification for mucociliary barrier function, potentially providing protection^23^. However, MUC2 has only been evaluated qualitatively; thus, quantitative and functional evaluations of clinicopathological features and MUC2 are subjects for future studies.

In the present study, MUC1, MUC6, and CD10 were negative in all cases. In contrast, Longman et al. reported that MUC1 expression was upregulated, with MUC1 mRNA localized to crypt abscesses in samples from patients with active UC^24^. Similarly, Gersemann et al. reported MUC1 mRNA to be upregulated in both inflamed and non-inflamed UC tissue samples compared with controls^25^. In our study, many patients had a relatively low degree of inflammation, which may explain why MUC1 expression was not detected. MUC6 has also been reported to be strongly associated with colorectal neoplastic lesions in patients with UC^18^. However, since the present study did not include colorectal neoplastic lesions, MUC6 expression was negative in all patients.

The mucus phenotype of the colon is dominated by MUC2, while gastric-type MUCs, such as MUC5AC (a mucus trait marker of the gastric foveolar epithelium type) and MUC6 (a marker of the pyloric gland type), are absent in normal colon tissue. Contrastingly, MUC5AC has been reported to be expressed in colon cancer and UC-related tumors, suggesting a link between colon mucosa tumorigenesis and MUC5AC expression. Mucous transformation to MUC5AC is common in UC-associated tumors, tumors and dysplasia of the serrated pathway, and Microsatellite instability and deficiencies in the mismatch repair machinery (MSI/dMMR) associated colorectal cancers^26−28^. Biopsy tissue samples in this study did not show dysplasia or UC-associated tumors; however, the samples were positive for MUC5AC, which was significantly more prevalent in the group with higher clinical, endoscopic, and histopathological activity scores, correlating with inflammation severity. Further research is needed to determine whether MUC5AC expression increases the incidence of tumors.

The mechanism underlying MUC5AC positivity and function in UC remains unclear. MUC expression is regulated by various factors such as intestinal microflora, IL-1β, TNF-α, Th2-type cytokines, and TTF1. Dysregulated differentiation of goblet cells, the main MUC-producing cells, also affects mucus phenotype, with goblet cell differentiation being regulated by the Hath1, KLF4, Notch, and Wnt pathways. These factors may cause abnormal synthesis and degradation of MUC, changing its glycoprotein structure (O-glycosylation, sulfation, sialylation, etc.), which may induce MUC5AC expression. Furthermore, MUC5AC expression has been reported to increase FUT8 levels in the mucosa of UC, which stimulates the production of a highly sticky mucus that increases the interaction between the microflora and the epithelium (mucus increases bacterial and toxin adhesion and penetration) and may induce further inflammation^29^. Conversely, MUC2 expression decreases in UC, and to compensate for the resulting loss of barrier and repair function, MUC5AC and TFF1 expression are promoted instead, as MUC5AC may affect mucosal healing^30,31^. Future studies focusing on the mechanism and function of MUC5AC induction are needed^32^.

Several reports have demonstrated a correlation between ultra-high magnification endoscopic findings and histopathological features^33,34^. However, the relationship between these endoscopic findings and mucus characteristics has not been fully investigated. Our findings suggest that ultra-high magnification endoscopy may provide insight into mucus-related mucosal changes in UC. Further studies are needed to clarify the functional significance of these findings.

In this study, MUC5AC expression was strongly associated with histopathological severity. It was also correlated with clinical and endoscopic severity, as well as EC classification, in univariate analysis. These findings may be explained by the limited sample size, which may have reduced the statistical power to detect significant associations. Given the established correlation between endoscopic and histopathological findings, further studies with larger sample sizes may help clarify the relationship between endoscopic findings and mucus phenotype. The biological significance of MUC5AC expression in UC remains unclear and warrants further investigation.

Furthermore, analysis conducted separately for inflammatory and non-inflammatory areas, as well as lesional and non-lesional areas, suggested that MUC5AC is more strongly associated with current inflammation than with past inflammation.

This study has several limitations. First, it was a single-center study with a relatively small sample size, and most cases had mild disease, which limited the analysis to qualitative evaluation of mucus characteristics. Second, mucus characteristics were assessed only at biopsy sites rather than throughout the entire colon. Third, the retrospective design may have introduced potential bias. In addition, this study used the Mayo score and MES, which were commonly used at the time of testing. Therefore, one limitation is that it did not use the Simple Clinical Colitis Activity Index (SCCAI) or Ulcerative Colitis Endoscopic Index of Severity (UCEIS), which have more evaluation items.

In conclusion, MUC5AC expression was strongly associated with histopathological severity in UC. It was also correlated with clinical and endoscopic severity, as well as ultra-high magnification endoscopic findings, in univariate analysis. Although the relationship between EC classification and mucus phenotype requires further clarification, these findings provide insight into mucus-related mucosal changes in UC. Further large-scale studies are needed to validate these findings.

## Supplementary Information

Below is the link to the electronic supplementary material.

Supplementary Material 1

## Data Availability

The datasets generated during and/or analysed during the current study are available from the corresponding author on reasonable request.
