# Supplementary material for: Association between mucus phenotypes, histopathological features, and ultra-high magnification endoscopic findings in ulcerative colitis
Source: Sci Rep. 2026 May 22;16:26692. doi: 10.1038/s41598-026-53592-3 (PMC13507151; doi:10.1038/s41598-026-53592-3)
Supplement: Supplementary file 1 [file 41598_2026_53592_MOESM1_ESM.pdf]

Supplementary Information

Title:

Association between Mucus Phenotypes, Histopathological Features, and Ultra-High Magnification Endoscopic Findings in Ulcerative Colitis

Takeshi Hashimoto[1)], Hiroki Kurumi[1)*], Shungo Kanetsuki[1)], Tsutomu Kanda[1)], Yuichiro Ikebuchi[1)], Koichiro Kawaguchi[1)], Kazuo Yashima[1)], Tomohiko Sakabe[2)], Yoshihisa Umekita[2)], Hajime Isomoto[1)]

1) Division of Gastroenterology and Nephrology, Department of Multidisciplinary Internal Medicine, Faculty of Medicine, Tottori University, Tottori, Japan
2) Department of Pathology, Faculty of Medicine, Tottori University, Tottori, Japan

*Correspondence: Hiroki Kurumi, Division of Gastroenterology and Nephrology, Department of Multidisciplinary Internal Medicine, Faculty of Medicine, Tottori University, Tottori, Japan (e-mail: kurumi_1022_1107@yahoo.co.jp).

<u>Supplementary Information</u>

1) We performed isotype control staining for each antibody used in this immunostaining and confirmed that the isotype control antibodies did not stain. As supplementary information, we present the images of the isotype control staining in Supplementary Figure S1.

Supplementary Figure S1.

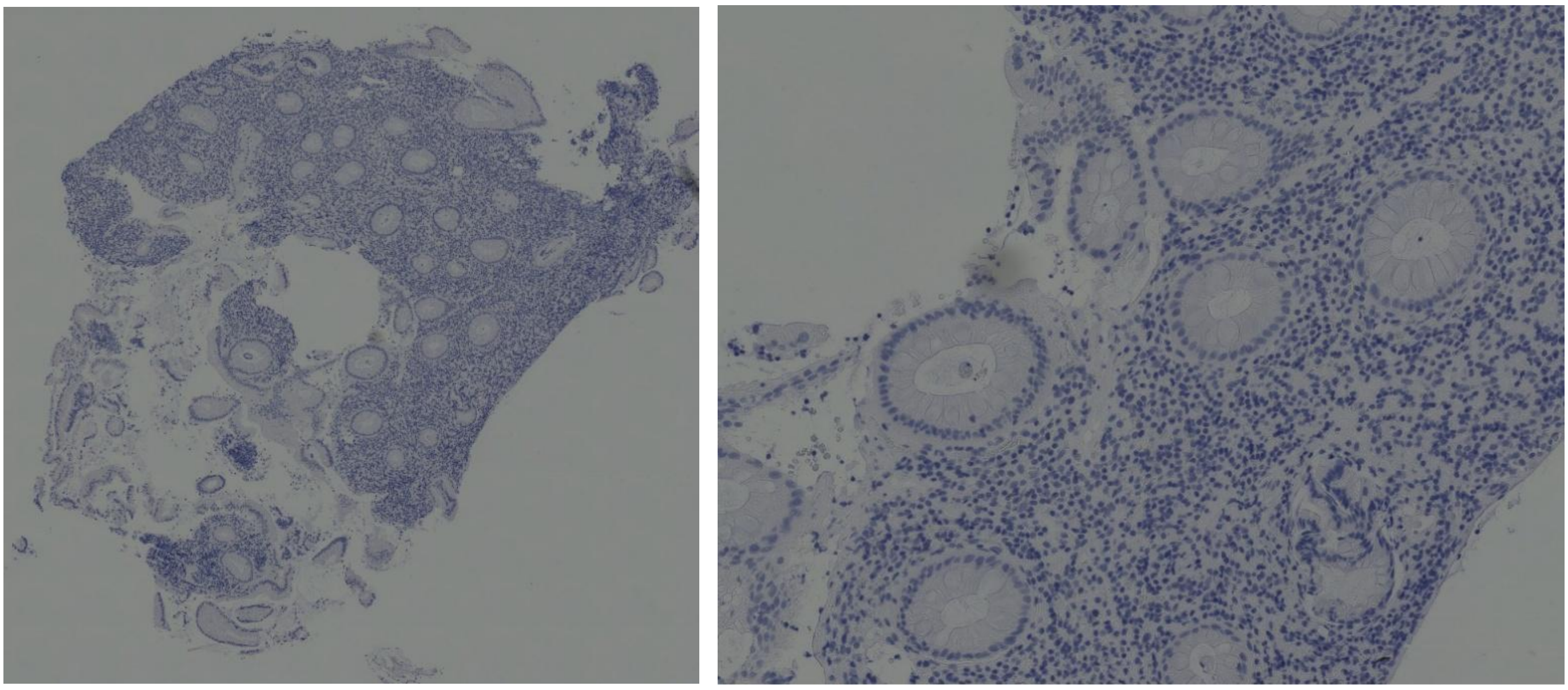

Figure Legends:

Supplementary Figure S1. Isotype control staining

Image of isotype control staining using a sample identical to the one shown in Figure 4 in the main text

Images were obtained using the microscope (BZ X710, KEYENCE Corp., Osaka, Japan) connected to a digital camera and saved as images.
